# Correction: Statistical context dictates the relationship between feedback-related EEG signals and learning

**DOI:** 10.7554/eLife.100526

**Published:** 2024-06-14

**Authors:** Matthew R Nassar, Rasmus Bruckner, Michael J Frank

**Keywords:** Human

Nassar MR, Bruckner R, Frank MJ. 2019. Statistical context dictates the relationship between feedback-related EEG signals and learning. *eLife* **8**:e46975. doi: 10.7554/eLife.46975.

Published 21 August 2019

The original text incorrectly stated the frequency range for bandpass filtering that was performed in our EEG pre-processing pipeline. This error occurred because we accidentally reported the filtering parameters associated with a separate EEG pre-processing pipeline optimized for time-frequency analyses instead of the one used for the ERP analyses that were analyzed in this paper. The actual bandpass filtering range was [0.05–15 hz] which emphasized lower frequency signal components in a manner consistent with our previous ERP publications (Collins et al 2014; Collins and Frank 2016; Collins and Frank, 2018). We thank Andrea Alamia and Xiaoqi Xu for identifying the discrepancy between our shared data (https://datadryad.org/stash/dataset/doi:10.5061/dryad.570pf8n) and our reported bandpass parameters and making us aware of the error.

Corrected text: Preprocessing was done manually in Matlab (Mathworks, Natick MA) using the EEGLAB toolbox (https://sccn.ucsd.edu/eeglab/index.php) as described previously (Collins and Frank, 2018) and included the following steps: (1) epoching and alignment to outcome onset, (2) epoch rejection by inspection, (3) channel removal and interpolation by inspection, (4) bandpass filtering [0.05–15 hz], (5) removal of blink and eye movement components using ICA.

Original text: Preprocessing was done manually in Matlab (Mathworks, Natick MA) using the EEGLAB toolbox (https://sccn.ucsd.edu/eeglab/index.php) as described previously (Collins and Frank, 2018) and included the following steps: (1) epoching and alignment to outcome onset, (2) epoch rejection by inspection, (3) channel removal and interpolation by inspection, (4) bandpass filtering [.1–50 hz], (5) removal of blink and eye movement components using ICA.]

The article has been corrected accordingly.

Collins, A.G.E. & Frank, M.J. (2016). Neural signature of hierarchically structured expectations predicts clustering and transfer of rule sets in reinforcement learning. Cognition, 152, 160-169.

Collins, A.G.E., Cavanagh, J.F. , Frank, M.J. 2014,. Human EEG uncovers latent generalizable task-set structure during learning. Journal of Neuroscience, 34, 4677-4685.

Collins AGE, Frank MJ. 2019. Within- and across-trial dynamics of human EEG reveal cooperative interplay between reinforcement learning and working memory. PNAS 115:2502-2507.

